# Full-Dose Distribution and Age–Dose Concordance of Suvorexant: A Retrospective Drug Utilization Study at a Japanese University Hospital

**DOI:** 10.3390/pharmacy14060127

**Published:** 2026-09-01

**Authors:** Shinya Kajiura, Nobukazu Ryu, Shingo Chikaoka, Yuta Yagi, Naohiko Nakamura, Atsushi Kato, Ryuji Hayashi

**Affiliations:** 1 Department of Medical Oncology and Palliative Medicine, Toyama University Hospital, Toyama 930-0194, Japan; naohiko@med.u-toyama.ac.jp (N.N.); hsayaka@med.u-toyama.ac.jp (R.H.); 2 Department of Hospital Pharmacy, Toyama University Hospital, Toyama 930-0194, Japan; ryu@med.u-toyama.ac.jp (N.R.); chikarin-tym@umin.ac.jp (S.C.); yyaaggii@med.u-toyama.ac.jp (Y.Y.); kato@med.u-toyama.ac.jp (A.K.)

**Keywords:** suvorexant, drug utilization study, dose selection, age–dose concordance, pharmacy practice, prescribing audit, medication-use evaluation

## Abstract

The Japanese label specifies suvorexant 20 mg once nightly for adults and 15 mg once nightly for older adults, but dose selection has not been characterized across all doses. We conducted a single-center retrospective drug-utilization study of outpatient prescriptions and inpatient medication orders at a Japanese university hospital during 2023–2025. Dose and prescription-date age were reconstructed, and patient-cluster bootstrap confidence intervals addressed repeated prescriptions. Among 3755 records, 15 mg accounted for 68.4%, 20 mg for 29.9%, and 10 mg and other calculable doses each for 0.9%. Of 3685 age-classifiable 15/20 mg records, 2529 were concordant with the operational age-based framework (68.6%; 95% CI 62.5–74.2). Non-concordance predominantly comprised 15 mg in patients younger than 65 years (841/1646; 51.1%); 20 mg in patients aged 65 years or older occurred in 315/2039 records (15.4%). Concordance was 65.0% in outpatient and 73.8% in inpatient records. Full-dose retention revealed uncommon 7.5- and 30-mg selections that a binary analysis would omit. Together, these findings provide a descriptive, single-center, full-dose, age- and setting-stratified basis for medication-use evaluation and targeted contextual review.

## 1. Introduction

Insomnia is managed through non-pharmacological strategies, particularly cognitive behavioral therapy for insomnia, and pharmacological treatment when clinically indicated. Guidelines emphasize treatment goals, comorbidity assessment, shared decision-making, and periodic reassessment rather than medication selection in isolation [1,2]. Comparative evidence shows that benefits and harms vary across hypnotic classes and treatment horizons, reinforcing the need to match therapy to the individual and to review continued use [3]. Dose selection is one modifiable component of medication management, especially in older adults, whose physiology, multimorbidity, and polypharmacy can alter exposure or susceptibility to central nervous system effects. Explicit medication criteria can assist screening in this population but are intended to support, rather than replace, clinical judgment [4]. Pharmacists contribute through medication assessment, counseling, interaction review, monitoring, and communication across care settings [5].

Suvorexant is a dual orexin receptor antagonist that blocks orexin-1 and orexin-2 receptor signaling, thereby reducing wake drive through a mechanism distinct from traditional sedative-hypnotics that enhance inhibitory neurotransmission [6,7]. Its pharmacology does not eliminate the importance of dose: systemic exposure rises with dose, the terminal half-life supports overnight activity, and accumulation and residual effects are relevant when treatment is repeated [8]. Controlled trials and later syntheses have evaluated sleep onset, sleep maintenance, somnolence, and other adverse effects across suvorexant regimens, while class-level comparative evidence continues to evolve [9]. These pharmacokinetic and pharmacodynamic features make the recorded dose a meaningful medication-use variable even when the database cannot determine the clinical outcome of a particular prescription.

Japan uses an age-specific labeled framework: 20 mg once nightly for adults and 15 mg once nightly for older adults [10]. The 20/15 mg non-elderly/elderly regimen was evaluated in phase III development, including pooled analyses, and older-adult evidence has been reported separately [11,12]. The framework therefore sits within a clinical-development program that explicitly incorporated age into dose assignment, although a label recommendation is not an absolute judgment of individual prescription appropriateness. The Japanese label also calls for consideration of 10 mg when a moderate CYP3A inhibitor is co-administered, consistent with primary pharmacokinetic evidence that CYP3A modulation changes suvorexant exposure [13]. By contrast, the United States label uses 10 mg as the starting dose and provides different interaction-based instructions [14].

Routine practice includes patients and treatment pathways that differ from trial populations. Previous hypnotic exposure, symptom trajectory, response to earlier doses, tolerability, concomitant medicines, and the purpose of a prescription may influence recorded selection. General implementation research also shows that recommendations interact with clinician knowledge, attitudes, resources, and workflow rather than being adopted mechanically [15]. Accordingly, a prescription that differs from a default dose can identify a decision requiring context, but dose and age alone cannot establish whether that decision was clinically justified. This distinction is central when administrative or pharmacy-system data lack the covariates needed for patient-level adjudication.

Drug-utilization research provides methods for describing how medicines are used and for linking quantitative patterns to qualitative assessment of determinants and consequences [16]. Within health-system pharmacy, medication-use evaluation is an ongoing, systematic, interdisciplinary process for examining and improving medication-use systems [17]. Prescription-data review can therefore evaluate implementation of a label-based decision rule without converting every deviation into an error classification. It may also separate implementation outcomes from clinical outcomes, a distinction needed when an audit measures recorded practice but not health effects [18]. Research on sleep-medicine prescribing further shows the value of scrutinizing what is recorded in routine systems, including dose instructions and age-related patterns [19].

A binary 15/20 mg concordance analysis can answer whether records align with the Japanese age-specific default, but it cannot describe all dose selection. Exact 10 mg and calculable 7.5- or 30-mg records would disappear from that denominator even though they remain informative for medication-use evaluation. Retaining them makes the primary denominator transparent, identifies non-default categories without judging them, and places the narrower concordance measure within the complete prescribing pattern. We therefore evaluated suvorexant dose selection across reconstructed age groups and outpatient and inpatient care settings at a Japanese university hospital. The primary objective was to describe the full-dose distribution of all included prescription records. The key secondary objective was to quantify age–dose concordance among age-classifiable exact 15/20 mg records, with sensitivity analyses addressing age-boundary uncertainty and repeated prescriptions.

## 2. Materials and Methods

### 2.1. Study Design and Setting

We conducted a single-center retrospective observational drug utilization study at Toyama University Hospital, a Japanese university hospital. The observation period was 1 January 2023 through 31 December 2025 and included outpatient and inpatient prescribing.

### 2.2. Data Source and Capture Point

Records were obtained from the hospital pharmacy information system. Outpatient data represented the final prescription recorded by the hospital; when an external pharmacy query resulted in a prescription change, the changed content was reflected in the hospital record. Inpatient data represented issued prescription orders. Available fields included prescription date, a pseudonymized patient identifier, recorded age, prescription category, drug formulation, tablet quantity, and related order fields.

### 2.3. Prescription Records and Care Setting

Outpatient records comprised hospital outpatient prescriptions and prescriptions intended for dispensing at community pharmacies. Inpatient records included regular, temporary, discharge, bridging, resumed-after-interruption, cross-department, and post-admission orders. Records of brought-in or discontinued medications were excluded. The primary unit of analysis was an individual prescription record, and repeated prescriptions for the same patient were retained.

### 2.4. Dose Reconstruction

The recorded tablet strength was multiplied by the authoritative per-administration tablet-quantity field to calculate milligrams per administration. A separate order-quantity field was used only for quality control. Doses were classified as exact 10 mg, exact 15 mg, exact 20 mg, other calculable dose, or classification indeterminate. The exact other calculable doses were retained in the audit output; no dose was inferred from total quantity alone.

### 2.5. Age Reconstruction

The recorded age represented completed years and months on 26 December 2025, not age on each prescription date. For each record, we retained the interval of possible dates of birth compatible with the recorded completed age. This interval was projected back to the prescription date to obtain a possible completed-age interval. Records whose maximum possible age was below 65 years were classified as definitely younger than 65; those whose minimum possible age was at least 65 were classified as definitely 65 or older; all others were age-boundary uncertain. We did not assign a single date of birth or an exact point age at prescription.

Age-boundary-uncertain records were retained in the primary full-dose distribution and reported as a separate category. Because only four such records were observed, they were omitted from the age-stratified graphical display to avoid presenting percentages from very small denominators with the same visual weight as the definitely age-classifiable strata. The main concordance analysis excluded uncertain-age records. Two boundary sensitivity scenarios assigned all uncertain records to younger than 65 or to 65 or older, respectively.

### 2.6. Outcomes

The primary outcome was the full-dose distribution among all included prescription records, overall and by reconstructed age group and care setting. The key secondary outcome was age–dose concordance among definitely age-classifiable records with an exact 15 or 20 mg dose. Concordance was defined as 20 mg for those definitely younger than 65 and 15 mg for those definitely 65 or older. Non-concordance was described by direction: 15 mg in those younger than 65 or 20 mg in those 65 or older.

### 2.7. Repeated-Prescription Sensitivity Analyses

A patient-day sensitivity dataset retained one record per patient, calendar date, and care setting; if multiple calculable doses occurred, the highest calculated dose was retained. A first-patient-day-within-setting analysis then retained the earliest patient-day separately for each setting. Patients could therefore contribute one outpatient and one inpatient first patient-day.

### 2.8. Statistical Analysis

Counts and percentages were reported for descriptive outcomes. Percentile 95% confidence intervals were calculated using 2000 patient-level cluster bootstrap replicates. Patients were resampled with replacement, and all prescription records for each selected patient across years and care settings were retained to preserve within-patient dependence.

Analyses were performed using Python version 3.12.13 (Python Software Foundation, Wilmington, DE, USA). The study was reported with reference to the STROBE statement [20].

### 2.9. Ethics

The study was conducted in accordance with the Declaration of Helsinki. The protocol was approved by the Institutional Review Board of Toyama University Hospital (Approval No. R2016138). Informed consent was waived under an institutional opt-out policy for retrospective research.

## 3. Results

### 3.1. Study Population

During the study period, we extracted 4069 suvorexant prescription records for 700 patients. We excluded 90 records of brought-in medications and 224 records of discontinued medications. The final analysis included 3755 prescription records for 656 patients, comprising 2217 outpatient and 1538 inpatient records. Of these, 1668 were from patients definitely younger than 65 years, 2083 were from patients definitely 65 years or older, and four records from four patients were age-boundary uncertain (Table 1).

### 3.2. Full-Dose Distribution

Exact 15 mg was the most frequent recorded dose, accounting for 2568/3755 prescription records (68.4%; 95% CI 62.1–74.8), followed by exact 20 mg in 1121/3755 records (29.9%; 95% CI 23.7–36.3). Exact 10 mg and other calculable doses each occurred in 33/3755 records (0.9%). The other calculable doses were 7.5 mg in 19 records and 30 mg in 14 records. No dose classification was indeterminate (Table 2).

**Figure 1:**
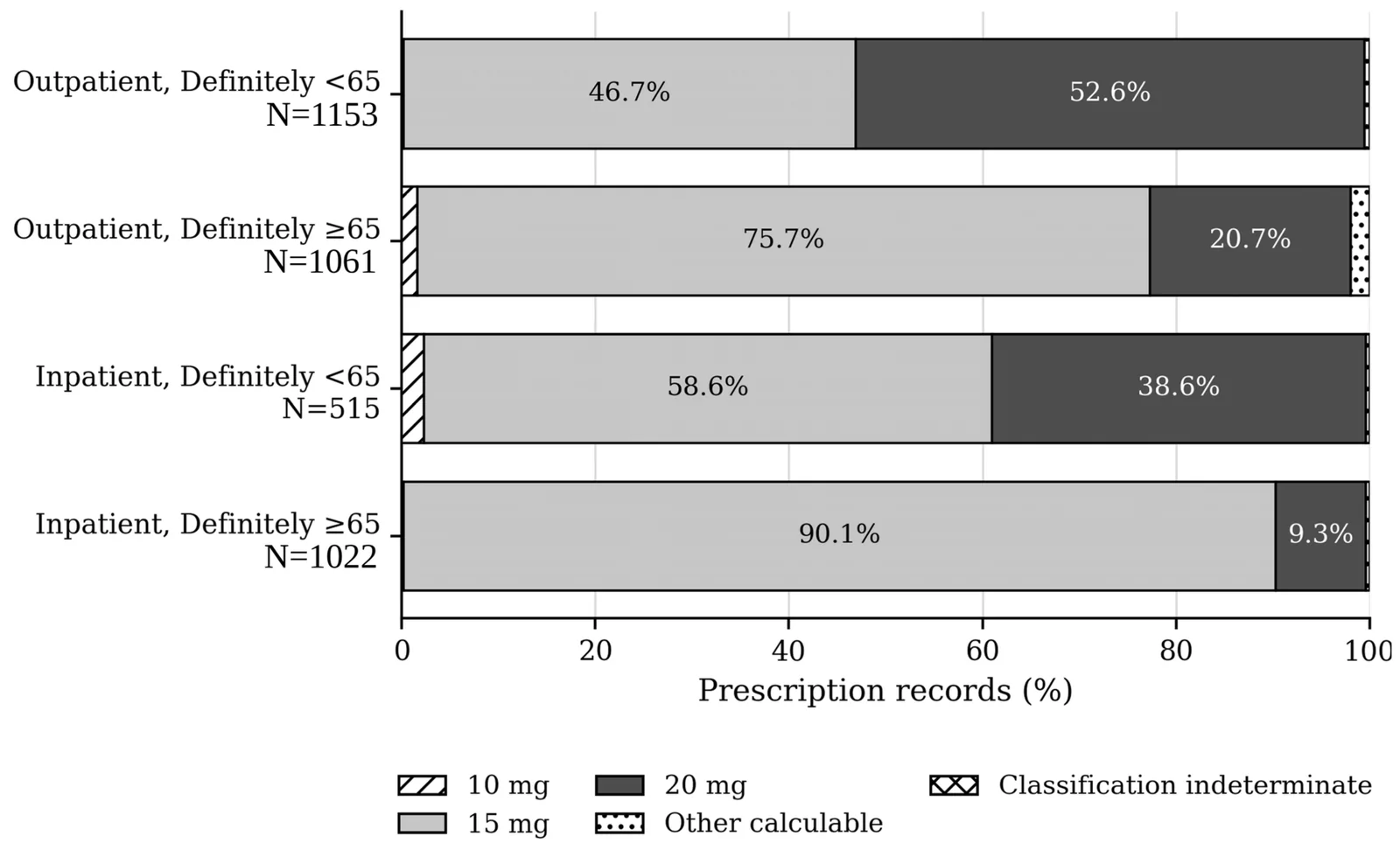
Full-dose distribution by canonical care setting and reconstructed age group. Bars show the percentage of prescription records classified as exact 10 mg, exact 15 mg, exact 20 mg, other calculable dose, or classification indeterminate. Age was reconstructed from completed age in years and months at 26 December 2025. Four age-boundary-uncertain records (three outpatient and one inpatient) were included in the overall full-dose distribution and Table 2 but omitted from the age-stratified graphical display because the very small denominators would give unstable percentages the same visual weight as the definitely age-classifiable strata. Denominators are displayed beside each stratum. The figure is descriptive; no *p*-value or causal setting comparison is reported.

The dose distribution differed descriptively across settings. Among outpatient prescription records, 15 mg accounted for 60.6% and 20 mg for 37.3%; among inpatient medication-order records, the corresponding proportions were 79.6% and 19.1%. Among records for patients definitely younger than 65 years, 15 mg and 20 mg accounted for 50.4% and 48.3%, respectively. Among records for patients definitely 65 years or older, they accounted for 82.8% and 15.1%. The full joint distribution, including the four age-boundary-uncertain records, is shown in Table 2; Figure 1 displays the four definitely age-classifiable setting-by-age strata.

Within the joint strata, 20 mg accounted for 52.6% of outpatient prescription records and 38.6% of inpatient medication-order records among patients definitely younger than 65 years. Among patients definitely 65 years or older, 20 mg accounted for 20.7% of outpatient prescription records and 9.3% of inpatient medication-order records. Exact 10 mg and other calculable doses remained uncommon in every joint stratum (Table 2; Figure 1).

### 3.3. Age–Dose Concordance

After restriction to exact 15/20 mg prescription records with definite age classification, 3685 records from 648 patients were eligible. Overall, 2529/3685 were concordant (68.6%; 95% CI 62.5–74.2), while 1156/3685 were non-concordant (31.4%; 95% CI 25.8–37.5). Concordance was 1409/2168 in outpatient records (65.0%; 95% CI 56.0–72.9) and 1120/1517 in inpatient records (73.8%; 95% CI 67.8–79.4) (Table 3).

By age group, 805/1646 records for patients definitely younger than 65 years were concordant (48.9%; 95% CI 38.3–58.4), and 1724/2039 records for patients definitely 65 years or older were concordant (84.6%; 95% CI 77.4–91.1). Non-concordance comprised 841 records with 15 mg in patients younger than 65 years and 315 records with 20 mg in patients aged 65 years or older. Four uncertain-age 15/20 mg records were excluded from the main denominator (Table 3).

### 3.4. Sensitivity Analyses

Assigning all four uncertain-age records to younger than 65 years yielded 2530/3689 concordant records (68.6%); assigning all four to 65 years or older yielded 2532/3689 (68.6%). The maximum absolute shift from the main estimate was less than 0.1 percentage point.

The patient-day dataset contained 3704 all-dose records; 2494/3635 eligible 15/20 mg patient-days were concordant (68.6%; 95% CI 62.5–74.2). The first-patient-day-within-setting analysis retained 745 all-dose records, of which 510/731 eligible records were concordant (69.8%; 95% CI 66.0–73.3). These sensitivity estimates are compared with the main prescription-record analysis in Table 4.

## 4. Discussion

### 4.1. Principal Findings

The primary full-dose analysis showed that 15 mg was the dominant recorded dose (2568/3755; 68.4%), followed by 20 mg (1121/3755; 29.9%); exact 10 mg and other calculable doses each represented 0.9%. The other calculated records comprised 19 prescriptions for 7.5 mg and 14 for 30 mg, and none was indeterminate. This all-dose view is the principal descriptive result because it retains every included prescription rather than defining the study population by the secondary 15/20 mg rule.

Within the key secondary denominator, 1156/3685 age-classifiable 15/20 mg records (31.4%) were non-concordant with the operational Japanese age-based framework; this classification does not by itself indicate clinically inappropriate prescribing. The predominant direction was 15 mg in patients younger than 65 years (841/1646; 51.1%; 95% CI 41.6–61.7); 20 mg in patients aged 65 years or older accounted for 315/2039 records (15.4%; 95% CI 8.9–22.6). Concordance was 65.0% in outpatient and 73.8% in inpatient records, with 20 mg more frequent in outpatient records within both age groups. Patient-day concordance was 68.6%, and first-patient-day concordance was 69.8%, supporting that repeated prescriptions did not materially produce the overall pattern (Table 4).

### 4.2. Why Full-Dose Analysis Matters

A binary concordance measure excludes doses other than exact 15 and 20 mg. If presented alone, 70 records—including every 10-, 7.5-, and 30-mg selection and the four age-boundary-uncertain 15/20 mg records—would be absent. Drug-utilization research favors transparent description before narrower evaluative rules are applied [16]. The primary denominator of 3755 answers a prior question from the concordance denominator of 3685: what full-dose pattern was recorded, rather than whether an eligible record matched a binary default.

The uncommon categories also matter operationally. The Japanese label provides a 10-mg context with moderate CYP3A inhibition, whereas 7.5 and 30 mg are non-default calculated amounts that may reflect tablet combinations, splitting, transition, or another undocumented decision [10]. None can be classified from dose alone as appropriate or inappropriate. Retaining the exact amounts nevertheless permits pharmacy teams to verify dose reconstruction, review denominators, and decide which records merit contextual inquiry [17].

### 4.3. Age-Based Dose Selection and Clinical-Trial Context

The Japanese 20/15 mg framework is grounded in a development program that assigned a lower regimen to participants aged 65 years or older. Pooled phase III analyses evaluated 20 mg in non-elderly and 15 mg in elderly patients over three months, while an elderly-specific pooled analysis characterized that age-defined regimen [11,12]. Long-term phase III evidence also assessed efficacy and tolerability during one year of treatment and after discontinuation, although the doses and populations of that trial should be interpreted within its protocol [21]. Drug-specific guidelines and reviews consequently place suvorexant among pharmacological options for insomnia while emphasizing the quality and limits of the underlying evidence [2,22].

Dose remains biologically relevant within that context. Rising-dose pharmacokinetic work demonstrated increasing exposure and characterized accumulation with repeated dosing [8]. Clinical-trial analyses have reported dose-related changes in patterns of wakefulness, and controlled next-morning driving studies separately examined 20/40 mg in non-elderly volunteers and 15/30 mg in healthy older volunteers [23,24,25]. Their mean findings do not remove concern for individual somnolence or residual effects, and healthy-volunteer results cannot adjudicate prescriptions in routine hospital populations. More recent network evidence compares dual orexin receptor antagonists and broader insomnia medicines at the treatment level, not the appropriateness of a recorded dose in an unmeasured clinical context [3,9].

The present study adds neither efficacy nor safety evidence to that trial literature. It asks whether recorded use implemented one locally defined age-based framework and how other doses were distributed. Thus, the 31.4% non-concordance estimate should not be interpreted as a trial-outcome signal, and the trial program should not be used to label every non-concordant record as erroneous. Its appropriate role is to explain why 20 and 15 mg were prespecified as the concordant categories and why dose selection is worth auditing.

### 4.4. Why Recorded Dose Selection May Deviate from the Default

Several plausible factors could produce a non-default recorded dose. A clinician might initiate cautiously, continue a dose that previously worked, respond to tolerability, account for concomitant therapy, or document a transitional prescription. General guideline-implementation research identifies knowledge, attitudes, and external constraints as possible influences, while geriatric prescribing guidance stresses that screening criteria must be applied thoughtfully [4,15]. Interpretive guidance likewise treats explicit criteria as prompts for review rather than substitutes for clinical judgment [26]. These possibilities explain why non-concordance is not synonymous with inappropriate prescribing; they were not observed or tested in this dataset.

Drug-interaction context is a concrete example of missing information. Suvorexant is predominantly metabolized through CYP3A, and phase I studies showed that inhibition increased exposure whereas induction reduced it [13]. Both Japanese and United States labels therefore provide interaction-specific dose instructions, although the recommended amounts differ by jurisdiction [10,14]. Because concomitant medicines were unavailable, this audit could not determine whether any 10-mg or other selection reflected a CYP3A consideration. The correct inference is that selected records may merit linked medication review, not that an interaction was present or that the dose was justified.

### 4.5. Japanese Real-World Context

Japanese post-marketing evidence shows that suvorexant has been used in broad routine-practice populations, including many older patients and people with comorbidities [27,28]. Other retrospective studies describe heterogeneous pathways such as switching from benzodiazepine receptor agonists or adding suvorexant to existing hypnotic treatment [29,30]. Together, these studies place dose selection within a Japanese routine-practice context characterized by diverse prior treatments and clinical backgrounds.

A nationwide Japanese claims analysis found that demographic and psychiatric characteristics were associated with selection of an orexin receptor antagonist rather than another hypnotic [31]. That question differs from dose selection among suvorexant records, and claims, post-marketing cohorts, switching studies, and repeated hospital prescriptions use different populations and denominators. Direct numerical comparison with our 68.6% concordance or 31.4% non-concordance would therefore be misleading. Together, the literature situates the audit within heterogeneous Japanese practice while leaving its percentages explicitly local.

### 4.6. Outpatient and Inpatient Patterns

The inpatient dataset contained a larger proportion of records from patients aged 65 years or older, which contributed to its greater overall concentration at 15 mg. However, a setting pattern also remained within age groups: 20 mg represented 52.6% of outpatient versus 38.6% of inpatient records among patients younger than 65 years and 20.7% versus 9.3% among those aged 65 years or older. Consequently, age composition alone does not describe the joint distribution. These within-age differences make care setting an informative dimension of medication-use evaluation and support separate review of outpatient and inpatient dose patterns. These setting differences are descriptive and should not be attributed to specific prescribing behaviors or clinical decision-making processes.

Outpatient records represented the final prescription stored by the hospital, whereas inpatient records represented issued orders. The audit therefore captured the dose distribution at the endpoint available for each medication-use pathway. Separate review can frame focused local questions about where age, dose, interaction status, and prescribing rationale are documented.

### 4.7. Pharmacy-Practice and Medication-Use Evaluation Implications

The study provides a repeatable pharmacy medication-use evaluation sequence: reconstruct the dose from authoritative fields; retain all positive calculable categories; display age and setting strata; apply a prespecified local rule to an eligible secondary denominator; and examine repetition and age uncertainty. This approach aligns with international drug-utilization methods and ASHP guidance on systematic, interdisciplinary medication-use evaluation [16,17]. It also keeps implementation measurement distinct from patient outcomes [18]. For practice, the output can guide a staged local process of contextual review, periodic audit and feedback, and locally tested prescribing-system improvement.

A health-system pharmacy service could use the framework for periodic audit and feedback, stratifying results by age and setting and reviewing a sample of non-default records alongside indication, previous response, tolerability, and concomitant medicines. Audit and feedback can change professional practice, but effects vary with baseline performance, recipient, format, and context [32]. Feedback is more actionable when repeated, focused on behavior, linked to an explicit target, and accompanied by a feasible response [33].

Electronic prescribing is another review point. Age-linked defaults, interaction alerts, and fields documenting the reason for a non-default dose could make the decision pathway more visible. Prior quasi-experimental work demonstrates that an electronic default can shift prescribing behavior, although it concerned generic medicines and does not establish the effect of a suvorexant dose default [34]. Any configuration should therefore be locally tested for usability, alert burden, exceptions, and unintended consequences. Pharmacist involvement may support data validation and contextual review, consistent with described roles in insomnia care, but the present observational study did not test an intervention [5].

### 4.8. International Relevance

The internationally transferable contribution is the audit method rather than a single dose rule: define the locally applicable reference, preserve the full-dose denominator, separate description from evaluation, address repeated records, stratify relevant contexts, and use deviations to prioritize review. Local adaptation is essential because the United States label begins at 10 mg, allows escalation to 20 mg, and uses different instructions with moderate CYP3A inhibitors, whereas Japan specifies the age-based 20/15 mg framework and consideration of 10 mg in the relevant interaction context [10,14]. The same method can therefore support medication-use evaluation across jurisdictions while retaining each jurisdiction’s labeling and practice context.

### 4.9. Strengths

Strengths include comprehensive capture across three consecutive years and both outpatient and inpatient settings; retention of all reconstructed doses, including 7.5 and 30 mg; use of the final recorded outpatient prescription; explicit resolution of canonical care setting; and transparent handling of age-boundary uncertainty. Patient-cluster confidence intervals preserved within-patient dependence, while patient-day and first-patient-day analyses tested whether prescription frequency dominated the concordance result. The full-dose-first hierarchy and explicit denominators make the audit reproducible and readily adaptable to repeat medication-use evaluation.

### 4.10. Limitations and Future Research

Several limitations constrain interpretation. This was a single-center study, so the percentages do not estimate national prevalence. The prescription records did not include indication, comorbidities, insomnia symptoms or severity, previous treatment response, tolerability, adverse events, concomitant medicines, CYP3A context, or clinician intent. Accordingly, the study could not identify why individual doses were selected, adjudicate prescription appropriateness, or evaluate efficacy, safety, continuation, or switching outcomes.

Age was reconstructed from completed years and months at a common reference date rather than exact birth dates, although only four records were boundary uncertain and the boundary analyses changed the estimate by less than 0.1 percentage point. Prescription-record analysis weights patients with frequent prescribing more heavily; clustering and the two reduced datasets addressed but did not erase this feature. Outpatient data represented the final hospital record rather than the original prescriber entry, whereas inpatient records were issued orders. External dispensing was not independently confirmed, and pharmacist queries were not identifiable. These unequal capture points preclude attributing the setting pattern to clinicians, pharmacists, or a particular workflow, quantifying a pharmacist effect, or comparing safety between settings. Future multicenter studies could link prescription records to concomitant medication, indication, documented rationale, symptoms, adverse effects, and outcomes, then evaluate whether a contextual review process is feasible and useful. Until such evidence is available, the present estimates should be used to structure inquiry, not to classify care.

## 5. Conclusions

Across 3755 suvorexant prescription records from three consecutive years at a Japanese university hospital, 15 mg was the most frequently recorded dose, while 10 mg, 7.5 mg, and 30 mg selections were uncommon but visible in the full-dose analysis. Among age-classifiable exact 15/20 mg records, 31.4% were non-concordant with the operational Japanese age-based framework, predominantly because 15 mg was recorded for patients younger than 65 years. Concordance and dose distributions also differed descriptively between outpatient and inpatient records, identifying setting-specific patterns for further review. At the study hospital, a full-dose-first, age- and setting-stratified medication-use evaluation may support periodic audit and prioritize prescriptions for contextual review. A potentially transferable contribution is a transparent audit framework that preserves non-default doses, applies locally relevant rules, accounts for repeated prescribing, and connects quantitative surveillance with focused clinical review.

## Figures and Tables

**Table 1:** Data source, canonical record flow, and analysis population. Outpatient records comprised hospital outpatient prescriptions and prescriptions intended for dispensing at community pharmacies. Inpatient records included regular, temporary, discharge, bridging, resumed-after-interruption, cross-department, and post-admission orders. Records of brought-in or discontinued medications were excluded.

Item	Result
Canonical master source files	2023, 2024, and 2025 all-setting master extracts
Observation period	1 January 2023 through 31 December 2025
Raw master records	4069
Excluded: brought-in medication	90
Excluded: medication stopped	224
Other excluded/unclassified	0
Included prescription records	3755
Outpatient prescription records	2217
Inpatient medication-order records	1538
Unique patients	656
Age-classifiable prescription records	3751
Age-boundary-uncertain prescription records	4 (4 patients)
Dose-calculable prescription records	3755
Classification indeterminate	0

**Table 2:** Full-dose distribution by reconstructed age group and canonical care setting. Data format: n/N (percentage; patient-cluster bootstrap 95% CI). Percentages may not sum to 100.0 because of rounding.

Stratum	N	10 mg	15 mg	20 mg	Other Calculable	Classification Indeterminate
**Overall**	**3755**	**33/3755 (0.9%;** **95% CI 0.2–1.7)**	**2568/3755 (68.4%;** **95% CI 62.1–74.8)**	**1121/3755 (29.9%;** **95% CI 23.7–36.3)**	**33/3755 (0.9%;** **95% CI 0.2–1.9)**	**0/3755 (0.0%;** **95% CI 0.0–0.0)**
Outpatient	2217	19/2217 (0.9%; 95% CI 0.1–1.9)	1344/2217 (60.6%; 95% CI 52.0–69.6)	827/2217 (37.3%; 95% CI 28.6–45.9)	27/2217 (1.2%; 95% CI 0.1–2.8)	0/2217 (0.0%; 95% CI 0.0–0.0)
Inpatient	1538	14/1538 (0.9%; 95% CI 0.1–2.0)	1224/1538 (79.6%; 95% CI 74.6–84.2)	294/1538 (19.1%; 95% CI 14.7–24.0)	6/1538 (0.4%; 95% CI 0.1–0.8)	0/1538 (0.0%; 95% CI 0.0–0.0)
Definitely <65 years	1668	14/1668 (0.8%; 95% CI 0.0–2.0)	841/1668 (50.4%; 95% CI 41.9–60.0)	805/1668 (48.3%; 95% CI 38.8–56.8)	8/1668 (0.5%; 95% CI 0.0–1.1)	0/1668 (0.0%; 95% CI 0.0–0.0)
Definitely ≥65 years	2083	19/2083 (0.9%; 95% CI 0.1–2.1)	1724/2083 (82.8%; 95% CI 75.6–89.0)	315/2083 (15.1%; 95% CI 8.9–22.4)	25/2083 (1.2%; 95% CI 0.0–3.0)	0/2083 (0.0%; 95% CI 0.0–0.0)
Age-boundary uncertain	4	0/4 (0.0%; 95% CI 0.0–0.0)	3/4 (75.0%; 95% CI 0.0–100.0)	1/4 (25.0%; 95% CI 0.0–100.0)	0/4 (0.0%; 95% CI 0.0–0.0)	0/4 (0.0%; 95% CI 0.0–0.0)
Outpatient × Definitely <65 years	1153	2/1153 (0.2%; 95% CI 0.0–0.6)	539/1153 (46.7%; 95% CI 35.9–59.1)	606/1153 (52.6%; 95% CI 40.3–63.4)	6/1153 (0.5%; 95% CI 0.0–1.4)	0/1153 (0.0%; 95% CI 0.0–0.0)
Outpatient × Definitely ≥65 years	1061	17/1061 (1.6%; 95% CI 0.0–3.8)	803/1061 (75.7%; 95% CI 65.1–86.2)	220/1061 (20.7%; 95% CI 10.8–31.5)	21/1061 (2.0%; 95% CI 0.0–5.2)	0/1061 (0.0%; 95% CI 0.0–0.0)
Outpatient × Age-boundary uncertain	3	0/3 (0.0%; 95% CI 0.0–0.0)	2/3 (66.7%; 95% CI 0.0–100.0)	1/3 (33.3%; 95% CI 0.0–100.0)	0/3 (0.0%; 95% CI 0.0–0.0)	0/3 (0.0%; 95% CI 0.0–0.0)
Inpatient × Definitely <65 years	515	12/515 (2.3%; 95% CI 0.0–5.3)	302/515 (58.6%; 95% CI 49.3–68.4)	199/515 (38.6%; 95% CI 29.4–47.8)	2/515 (0.4%; 95% CI 0.0–1.3)	0/515 (0.0%; 95% CI 0.0–0.0)
Inpatient × Definitely ≥65 years	1022	2/1022 (0.2%; 95% CI 0.0–0.7)	921/1022 (90.1%; 95% CI 85.9–93.7)	95/1022 (9.3%; 95% CI 5.6–13.6)	4/1022 (0.4%; 95% CI 0.0–0.9)	0/1022 (0.0%; 95% CI 0.0–0.0)
Inpatient × Age-boundary uncertain	1	0/1 (0.0%; 95% CI 0.0–0.0)	1/1 (100.0%; 95% CI 100.0–100.0)	0/1 (0.0%; 95% CI 0.0–0.0)	0/1 (0.0%; 95% CI 0.0–0.0)	0/1 (0.0%; 95% CI 0.0–0.0)

**Table 3:** Age–dose concordance among age-classifiable 15/20 mg prescription records. Definition: Concordance was defined as 20 mg for patients definitely younger than 65 years and 15 mg for patients definitely 65 years or older. Four age-boundary-uncertain 15/20 mg records were excluded from the main denominator.

Stratum	Eligible N	Patients	Concordant	Non-Concordant	<65: 15 mg	≥65: 20 mg
**Overall**	**3685**	**648**	**2529/3685 (68.6%;** **95% CI 62.5–74.2)**	**1156/3685 (31.4%;** **95% CI 25.8–37.5)**	**841/3685 (22.8%;** **95% CI 18.0–28.1)**	**315/3685 (8.5%;** **95% CI 4.8–12.9)**
Outpatient	2168	329	1409/2168 (65.0%; 95% CI 56.0–72.9)	759/2168 (35.0%; 95% CI 27.1–44.0)	539/2168 (24.9%; 95% CI 18.0–32.4)	220/2168 (10.1%; 95% CI 5.0–16.7)
Inpatient	1517	406	1120/1517 (73.8%; 95% CI 67.8–79.4)	397/1517 (26.2%; 95% CI 20.6–32.2)	302/1517 (19.9%; 95% CI 14.9–25.6)	95/1517 (6.3%; 95% CI 3.8–9.3)
Definitely <65 years	1646	279	805/1646 (48.9%; 95% CI 38.3–58.4)	841/1646 (51.1%; 95% CI 41.6–61.7)	841/1646 (51.1%; 95% CI 41.6–61.7)	0/1646 (0.0%; 95% CI 0.0–0.0)
Definitely ≥65 years	2039	372	1724/2039 (84.6%; 95% CI 77.4–91.1)	315/2039 (15.4%; 95% CI 8.9–22.6)	0/2039 (0.0%; 95% CI 0.0–0.0)	315/2039 (15.4%; 95% CI 8.9–22.6)

**Table 4:** Age–dose concordance in prescription-record, patient-day, and first-patient-day analyses. The prescription-record estimates are repeated from Table 3 for direct comparison. Patient-day and first-patient-day datasets were defined as described in the Section 2. Percentile 95% confidence intervals were calculated using patient-level cluster bootstrap.

Analysis Unit	Setting	Concordant *n*/N	Concordance, % (95% CI)
**Prescription record (main)**	**Overall**	**2529/3685**	**68.6 (62.5–74.2)**
Prescription record (main)	Outpatient	1409/2168	65.0 (56.0–72.9)
Prescription record (main)	Inpatient	1120/1517	73.8 (67.8–79.4)
**Patient-day**	**Overall**	**2494/3635**	**68.6 (62.5–74.2)**
Patient-day	Outpatient	1409/2168	65.0 (56.2–72.5)
Patient-day	Inpatient	1085/1467	74.0 (68.1–79.3)
**First-patient-day**	**Overall**	**510/731**	**69.8 (66.0–73.3)**
First-patient-day	Outpatient	207/327	63.3 (57.8–68.4)
First-patient-day	Inpatient	303/404	75.0 (70.6–78.9)

## Data Availability

Subject to institutional approval, the de-identified dataset supporting this study’s findings is stored on secure institutional servers and is not publicly available because of patient privacy and institutional policy. Additional data may be shared by the corresponding author upon reasonable request, pending institutional approval and data-sharing agreements.

## References

[1] Qaseem A., Kansagara D., Forciea M.A., Cooke M., Denberg T.D. (2016). Management of Chronic Insomnia Disorder in Adults: A Clinical Practice Guideline From the American College of Physicians. Ann. Intern. Med..

[2] Sateia M.J., Buysse D.J., Krystal A.D., Neubauer D.N., Heald J.L. (2017). Clinical Practice Guideline for the Pharmacologic Treatment of Chronic Insomnia in Adults: An American Academy of Sleep Medicine Clinical Practice Guideline. J. Clin. Sleep Med..

[3] De Crescenzo F., D’Alò G.L., Ostinelli E.G., Ciabattini M., Di Franco V., Watanabe N., Kurtulmus A., Tomlinson A., Mitrova Z., Foti F. (2022). Comparative effects of pharmacological interventions for the acute and long-term management of insomnia disorder in adults: A systematic review and network meta-analysis. Lancet.

[4] (2023). American Geriatrics Society 2023 updated AGS Beers Criteria^®^ for potentially inappropriate medication use in older adults. J. Am. Geriatr. Soc..

[5] Basheti M.M., Gordon C., Grunstein R., Saini B. (2025). Exploring the pharmacist role in insomnia management and care provision: A scoping review. J. Am. Pharm. Assoc..

[6] Cox C.D., Breslin M.J., Whitman D.B., Schreier J.D., McGaughey G.B., Bogusky M.J., Roecker A.J., Mercer S.P., Bednar R.A., Lemaire W. (2010). Discovery of the dual orexin receptor antagonist [(7R)-4-(5-chloro-1,3-benzoxazol-2-yl)-7-methyl-1,4-diazepan-1-yl][5-methyl-2-(2H-1,2,3-triazol-2-yl)phenyl]methanone (MK-4305) for the treatment of insomnia. J. Med. Chem..

[7] Rhyne D.N., Anderson S.L. (2015). Suvorexant in insomnia: Efficacy, safety and place in therapy. Ther. Adv. Drug Saf..

[8] Yee K.L., McCrea J., Panebianco D., Liu W., Lewis N., Cabalu T., Ramael S., Wrishko R.E. (2018). Safety, Tolerability, and Pharmacokinetics of Suvorexant: A Randomized Rising-Dose Trial in Healthy Men. Clin. Drug Investig..

[9] Kishi T., Ikuta T., Citrome L., Sakuma K., Hatano M., Hamanaka S., Nishii Y., Iwata N. (2025). Comparative efficacy and safety of daridorexant, lemborexant, and suvorexant for insomnia: A systematic review and network meta-analysis. Transl. Psychiatry.

[10] Pharmaceuticals and Medical Devices Agency Belsomra Tablets 10 mg/15 mg/20 mg [Japanese Electronic Package Insert]. https://www.pmda.go.jp/PmdaSearch/iyakuDetail/ResultDataSetPDF/170050_1190023F1024_1_19.

[11] Herring W.J., Connor K.M., Snyder E., Snavely D.B., Zhang Y., Hutzelmann J., Matzura-Wolfe D., Benca R.M., Krystal A.D., Walsh J.K. (2016). Suvorexant in Patients with Insomnia: Pooled Analyses of Three-Month Data from Phase-3 Randomized Controlled Clinical Trials. J. Clin. Sleep Med..

[12] Herring W.J., Connor K.M., Snyder E., Snavely D.B., Zhang Y., Hutzelmann J., Matzura-Wolfe D., Benca R.M., Krystal A.D., Walsh J.K. (2017). Suvorexant in Elderly Patients with Insomnia: Pooled Analyses of Data from Phase III Randomized Controlled Clinical Trials. Am. J. Geriatr. Psychiatry.

[13] Wrishko R.E., McCrea J.B., Yee K.L., Liu W., Panebianco D., Mangin E., Chakravarthy M., Martinez-Cantarin M.P., Kraft W.K. (2019). Effect of CYP3A Inhibition and Induction on the Pharmacokinetics of Suvorexant: Two Phase I, Open-Label, Fixed-Sequence Trials in Healthy Subjects. Clin. Drug Investig..

[14] U.S. Food and Drug Administration BELSOMRA (Suvorexant) Tablets, Prescribing Information. https://www.accessdata.fda.gov/drugsatfda_docs/label/2025/204569s010lbl.pdf.

[15] Cabana M.D., Rand C.S., Powe N.R., Wu A.W., Wilson M.H., Abboud P.A., Rubin H.R. (1999). Why don’t physicians follow clinical practice guidelines? A framework for improvement. JAMA.

[16] World Health Organization (2003). Introduction to Drug Utilization Research.

[17] Afanasjeva J., Burk M., Cunningham F.F., Fanikos J., Gabay M., Hayes G.J., Masters P.L., Rodriguez R., Sinnett M.J. (2021). ASHP Guidelines on Medication-Use Evaluation. Am. J. Health Syst. Pharm..

[18] Proctor E., Silmere H., Raghavan R., Hovmand P., Aarons G., Bunger A., Griffey R., Hensley M. (2011). Outcomes for implementation research: Conceptual distinctions, measurement challenges, and research agenda. Adm. Policy Ment. Health.

[19] Eronen S.-T., Kurko T., Kivelä S.-L., Paunio T., Airaksinen M., Rantamäki T. (2024). Sleep medicines are often prescribed for older adults (≥75 years) without appropriate dosing instructions: A nationwide retrospective register study in Finland. Acta Psychiatr. Scand..

[20] von Elm E., Altman D.G., Egger M., Pocock S.J., Gøtzsche P.C., Vandenbroucke J.P. (2007). The Strengthening the Reporting of Observational Studies in Epidemiology (STROBE) statement: Guidelines for reporting observational studies. Lancet.

[21] Michelson D., Snyder E., Paradis E., Chengan-Liu M., Snavely D.B., Hutzelmann J., Walsh J.K., Krystal A.D., Benca R.M., Cohn M. (2014). Safety and efficacy of suvorexant during 1-year treatment of insomnia with subsequent abrupt treatment discontinuation: A phase 3 randomised, double-blind, placebo-controlled trial. Lancet Neurol..

[22] Tampi R.R., Manikkara G., Balachandran S., Taparia P., Hrisko S., Srinivasan S., Tampi D.J. (2018). Suvorexant for insomnia in older adults: A perspective review. Drugs Context.

[23] Svetnik V., Snyder E.S., Tao P., Scammell T.E., Roth T., Lines C., Herring W.J. (2018). Insight Into Reduction of Wakefulness by Suvorexant in Patients With Insomnia: Analysis of Wake Bouts. Sleep.

[24] Vermeeren A., Sun H., Vuurman E.F., Jongen S., Van Leeuwen C.J., Van Oers A.C., Palcza J., Li X., Laethem T., Heirman I. (2015). On-the-Road Driving Performance the Morning after Bedtime Use of Suvorexant 20 and 40 mg: A Study in Non-Elderly Healthy Volunteers. Sleep.

[25] Vermeeren A., Vets E., Vuurman E.F., Van Oers A.C., Jongen S., Laethem T., Heirman I., Bautmans A., Palcza J., Li X. (2016). On-the-road driving performance the morning after bedtime use of suvorexant 15 and 30 mg in healthy elderly. Psychopharmacology.

[26] Steinman M.A., Beizer J.L., DuBeau C.E., Laird R.D., Lundebjerg N.E., Mulhausen P. (2015). How to Use the American Geriatrics Society 2015 Beers Criteria-A Guide for Patients, Clinicians, Health Systems, and Payors. J. Am. Geriatr. Soc..

[27] Asai Y., Sano H., Miyazaki M., Iwakura M., Maeda Y., Hara M. (2019). Suvorexant (Belsomra(^®^) Tablets 10, 15, and 20 mg): Japanese Drug-Use Results Survey. Drugs R D..

[28] Takeuchi Y., Sano H., Asai Y., Miyazaki M., Iwakura M., Maeda Y. (2020). Real-world evidence of the safety and efficacy profile of suvorexant in elderly patients with insomnia: A sub-analysis of the post-marketing drug-use results survey in Japan. Curr. Med. Res. Opin..

[29] Hatano M., Kamei H., Inagaki R., Matsuzaki H., Hanya M., Yamada S., Iwata N. (2018). Assessment of Switching to Suvorexant versus the Use of Add-on Suvorexant in Combination with Benzodiazepine Receptor Agonists in Insomnia Patients: A Retrospective Study. Clin. Psychopharmacol. Neurosci..

[30] Tachibana M., Kanahara N., Oda Y., Hasegawa T., Kimura A., Iyo M. (2024). A retrospective clinical practice study comparing the usefulness of dual-orexin receptor antagonists and a melatonin receptor agonist in patients switching from long-term benzodiazepine receptor agonists. J. Clin. Sleep Med..

[31] Okuda S., Qureshi Z.P., Yanagida Y., Ito C., Homma Y., Tokita S. (2023). Factors Associated with Prescriptions for an Orexin Receptor Antagonist Among Japanese Patients with Insomnia: Analysis of a Nationwide Japanese Claims Database. Drugs—Real World Outcomes.

[32] Ivers N., Yogasingam S., Lacroix M., Brown K.A., Antony J., Soobiah C., Simeoni M., Willis T.A., Crawshaw J., Antonopoulou V. (2026). Audit and feedback: Effects on professional practice. Cochrane Database Syst. Rev..

[33] Brehaut J.C., Colquhoun H.L., Eva K.W., Carroll K., Sales A., Michie S., Ivers N., Grimshaw J.M. (2016). Practice Feedback Interventions: 15 Suggestions for Optimizing Effectiveness. Ann. Intern. Med..

[34] Patel M.S., Day S., Small D.S., Howell J.T., Lautenbach G.L., Nierman E.H., Volpp K.G. (2014). Using default options within the electronic health record to increase the prescribing of generic-equivalent medications: A quasi-experimental study. Ann. Intern. Med..

